# Supplementary material for: The Impact of Cytokines on the Health-Related Quality of Life in Patients with Systemic Lupus Erythematosus
Source: J Clin Med. 2019 Jun 15;8(6):857. doi: 10.3390/jcm8060857 (PMC6617124; doi:10.3390/jcm8060857)
Supplement: Supplementary file 1 [file jcm-08-00857-s001.pdf]

Supplementary Table S1. Spearman rank correlation coefficients (Rs) for relation between SF36 domain and summary scores with clinical findings and disease activity and presence of organ damage.

	Physical Scales				Mental scales				PCS	MCS
	PF	RP	BP	GH	VT	SF	RE	MH		
Demographics										
Age visit	**-0.44****	-0.22	-0.20	0.00	-0.09	-0.23	**-0.29***	-0.09	-0.27	**-0.31***
Time since diagnosis	-0.01	0.02	0.14	0.15	0.07	0.10	**-0.29***	-0.16	0.11	-0.12
Female	-0.19	0.02	-0.15	-0.16	-0.14	-0.11	0.07	-0.04	-0.18	-0.07
Schooling	0.26	0.18	0.13	0.26	0.06	0.04	0.21	0.04	0.27	0.13
Required social security	0.19	-0.08	-0.21	-0.03	-0.12	-0.07	0.02	-0.08	-0.08	-0.05
BMI	0.10	0.15	0.01	0.24	**0.32***	**0.29***	0.11	0.13	0.15	0.24
Smoker	-0.13	0.00	-0.27	**-0.33***	-0.21	-0.18	0.04	-0.05	-0.20	-0.08
No cigarettes daily	-0.03	-0.09	0.12	-0.21	-0.18	-0.24	-0.01	**-0.48****	-0.11	-0.13
Comorbidity										
Cardiovascular events	-0.21	-0.14	0.13	-0.15	-0.06	-0.03	-0.11	-0.08	-0.06	-0.08
Stroke	-0.26	-0.03	-0.08	-0.07	-0.11	-0.09	-0.10	-0.03	-0.07	0.03
High blood pressure	**-0.29***	-0.05	0.12	0.01	0.12	-0.07	0.05	0.09	-0.05	0.04
Mental Health Issues	-0.02	-0.04	-0.03	-0.02	-0.01	-0.04	0.05	-0.06	-0.02	-0.02
Neurological Deficits	-0.20	-0.19	0.05	-0.05	-0.08	-0.11	-0.02	-0.13	-0.11	-0.12
Gastrointestinal issues	-0.22	-0.13	-0.19	-0.03	-0.22	-0.14	-0.21	-0.26	-0.20	**-0.31***
Thyroid issues	-0.12	-0.19	-0.06	-0.06	0.05	-0.03	**-0.29***	-0.11	-0.15	-0.22
Medication usage										
Prednisone	-0.17	-0.06	0.05	-0.08	-0.15	-0.16	-0.09	**-0.36****	-0.11	-0.24
Prednisone dose (mg)	-0.27	-0.16	0.04	-0.13	-0.17	-0.25	-0.04	**-0.35***	-0.20	-0.26
Hydroxychloroquine	0.03	0.19	0.01	0.00	0.08	0.04	-0.02	-0.21	0.09	0.03
Immunosuppressants	-0.09	-0.19	-0.11	-0.18	-0.10	-0.14	0.02	-0.04	-0.22	-0.12
NSAID	0.03	0.10	-0.21	-0.02	-0.07	-0.03	0.19	0.24	-0.01	0.15
Anticoagulants	-0.03	-0.22	-0.02	-0.17	-0.09	-0.07	-0.20	-0.11	-0.09	-0.16
Antihypertensive	-0.19	0.02	0.22	-0.06	-0.03	0.04	-0.17	-0.08	0.01	-0.11
Statins	-0.12	-0.20	0.00	-0.15	-0.14	-0.06	-0.14	-0.13	-0.11	-0.16
Anti-resorptive	**-0.49****	-0.13	-0.11	**-0.28***	-0.08	**-0.33***	0.07	-0.09	**-0.32***	-0.14

Mood regulators	-0.04	0.00	-0.07	0.03	0.06	0.03	0.13	0.02	-0.02	0.07
Analgesic	-.318*	-0.24	-0.37**	-0.27	-0.08	-0.30*	0.25	0.23	-0.39**	0.03
Fish oil regularly	-0.01	-0.04	-0.26	0.02	0.12	0.05	0.25	0.36**	-0.07	0.26
Laboratory findings										
Anti-dsDNA pos	0.01	-0.01	0.01	0.14	0.19	0.01	-0.09	-0.05	0.03	-0.03
Hypocomplementemia	-0.23	0.06	-0.06	-0.03	-0.06	-0.27	-0.04	0.05	-0.08	-0.14
Proteinuria>0.5 gr	0.10	0.07	0.17	0.03	0.09	0.22	-0.03	0.04	0.13	0.09
Haemoglobin(g/dL)	-0.01	-0.07	-0.11	-0.08	-0.17	-0.11	-0.07	-0.05	-0.08	-0.15
WBC	-0.29*	-0.25	-0.14	-0.33*	-0.05	-0.17	0.08	-0.13	-0.28*	-0.05
Lymphocytes	0.00	-0.07	-0.06	0.01	-0.10	0.11	-0.07	-0.03	-0.01	-0.02
ESR	0.17	-0.10	0.09	0.02	0.28*	0.15	-0.16	0.05	0.07	0.08
Hs-CRP	0.03	-0.18	0.01	-0.02	0.28*	0.03	0.15	0.01	-0.05	0.16
Creatinine (umol/l)	0.16	0.21	0.02	0.14	0.03	0.21	-0.10	0.14	0.15	0.03
Disease severity										
SLEDAI-2K score	-0.18	-0.09	-0.10	-0.22	-0.12	-0.31*	-0.06	-0.26	-0.21	-0.22
Patient VAS	-0.41**	-0.38**	-0.63**	-0.47**	-0.46**	-0.47**	-0.11	-0.21	-0.64**	-0.37**
Physicians VAS	-0.27	-0.23	-0.37**	-0.48**	-0.33*	-0.42**	0.02	-0.10	-0.46**	-0.22
Organ damage present	-0.42**	-0.12	0.03	-0.25	0.01	-0.33*	-0.02	-0.15	-0.20	-0.13

physical function (PF), role limitations due to physical problems (role physical, RP), bodily pain (BP), general health (GH), vitality (VT), social function (SF), role limitations due to emotional problems (role emotional, RE), and mental health (MH), PCS physical component summary score, MCS mental component summary score. * indicates p<0.05, ** indicates p<0.01.
